# Gambogenic acid inhibits fibroblast growth factor receptor signaling pathway in erlotinib-resistant non-small-cell lung cancer and suppresses patient-derived xenograft growth

**DOI:** 10.1038/s41419-018-0314-6

**Published:** 2018-02-15

**Authors:** Linfeng Xu, Xiaoxiao Meng, Naihan Xu, Wenwei Fu, Hongsheng Tan, Li Zhang, Qianjun Zhou, Jianan Qian, Shiwei Tu, Xueting Li, Yuanzhi Lao, Hongxi Xu

**Affiliations:** 1 School of Pharmacy, Shanghai University of Traditional Chinese Medicine, 201203 Shanghai, P.R. China; 2 Engineering Research Center of Shanghai Colleges for TCM New Drug Discovery, 201203 Shanghai, P.R. China; 3 Shanghai Chempartner Co., Ltd, 201203 Shanghai, P.R. China; 4 Key Lab in Healthy Science and Technology, Division of Life Science, Graduate School at Shenzhen, Tsinghua University, Room L410, Building L. Tsinghua Campus, 518055 Shenzhen, P.R. China; 5 Shanghai Lung Cancer Center, Shanghai Jiao Tong University School of Medicine (SJTUSM), Shanghai, P.R. China

## Abstract

Erlotinib resistance causes a high degree of lethality in non-small-cell lung cancer (NSCLC) patients. The high expression and activation of several receptor tyrosine kinases, such as JAK/STAT3, c-Met, and EGFR, play important roles in drug resistance. The development of tyrosine kinase inhibitors is urgently required in the clinic. Our previous study found that Gambogenic acid (GNA), a small molecule derived from the traditional Chinese medicine herb gamboge, induced cell death in several NSCLC cell lines through JAK/STAT3 inhibition. In this study, we investigated the mechanism of action of GNA in erlotinib-resistant NSCLC and patient-derived cells. The inhibition of GNA on FGFR signaling pathway was examined using biochemical kinase assays. NSCLC cell lines (HCC827, HCC827-Erlotinib-resistant, and H1650) and primary cells from patients with NSCLC with clinical resistance to erlotinib were treated with GNA, erlotinib, or their combination. Both kinase assays and cell- based assays showed that GNA inhibits the phosphorylation of multiple kinases in FGFR signaling pathway in NSCLC. The combination of GNA and erlotinib significantly attenuates the tumor growth of HCC827 and erlotinib-resistant HCC827 xenografts with low toxicity. Importantly, GNA significantly suppresses tumor growth in a lung patient-derived xenograft (PDX) model with FGFR fusion and low EGFR expression. Our findings provide preclinical evidence for using GNA as an FGFR signaling pathway inhibitor to overcome erlotinib resistance in NSCLC treatment or to enhance erlotinib efficacy when used as a combined administration.

## Introduction

Non-small-cell lung cancer (NSCLC) is the predominant form of lung cancer, which is a leading cause of cancer-related mortality and has very low 5-year survival rate^1–3^. NSCLCs are heterogeneous diseases, and among which, a variety of mutated genes have been identified, including tyrosine protein kinases. Epidermal growth factor receptor (EGFR) is a member of the ErbB family of transmembrane receptor tyrosine kinases, which is involved in signal transduction pathways regulating proliferation, apoptosis, epithelial−mesenchymal transition, and metastasis. Erlotinib, an EGFR inhibitor, was approved by the Food and Drug Administration (FDA) as a first-line treatment for metastatic NSCLC patients with EGFR mutations^4^. However, the relatively rapid emergence of resistance to erlotinib substantially limits its overall therapeutic benefit, reflecting a nonuniform response to treatment across all tumor cells within a patient and a role for intratumor heterogeneity. Drug resistance mechanisms have been elucidated in diverse cancer types, including NSCLC. Specifically, the hyperactivation of various protein kinases, such as c-Met, Akt, and Erk, is reported to contribute to the NSCLC drug resistance^5^. For instance, EGFR interacts with c-Met, and the activation of EGFR promotes the phosphorylation of c-Met^6–8^. Thus, a combined treatment with an EGFR inhibitor and c-Met inhibitors in NSCLC could increase the efficacy of the single treatment.

Fibroblast growth factor receptor (FGFR) is also frequently mutated in NSCLC and is considered a potential therapeutic target^9^. Interestingly, FGFR family proteins not only contribute to tumorigenesis but also the response to several chemotherapeutics, including erlotinib, gefitinib, and trametinib^10^. Recent evidence suggests that FGFR plays an important role in inducing drug resistance in NSCLC^11^. For instance, the enhanced expression of FGFR1 and FGF2 causes lung cancer cells to escape from afatinib (a pan-EGFR kinase inhibitor) treatment, suggesting that the FGFR signaling pathway could compensate for the loss of the EGFR-driven signal^12^. Thus, the development of FGFR inhibitors and how to precisely apply FGFR inhibitors are hot topics in cancer research.

Natural compounds are abundant pools for new drug development. GNA is a caged xanthone extracted from the traditional Chinese medicine gamboges and is a derivative of opening the pyran ring of gambogic acid (GA). Of note, GA was approved by the Chinese Food and Drug Administration for a phase II clinical trial in solid cancer therapy. In the preclinical studies, GA exerts a synergetic effect with cisplatin in NSCLC and overcomes imatinib resistance by enhancing Bcr-Abl downregulation in chronic myeloid leukemia cells^13,14^. In addition, some of the GA protein targets were identified by various techniques^15,16^. By using a proteomics approach, we identified that both GA and GNA targeted stathmin (STMN1) on hepatocellular carcinoma and enhance chemotherapy drug efficacy in an animal model^17^. In addition, a recent study indicated a potential role for GNA to increase the sensitivity of breast cancer to adriamycin by suppressing the PTEN/PI3K/AKT pathway^18^. In a cell proliferation screening assay of NCI-60 cell lines, we found that GNA and its variants exert a high efficacy on multiple cell lines, including NSCLC, and one of the protein targets was the STAT3/JAK signaling pathway^19^.

In this study, we investigated the anti-proliferative effects of GNA in NSCLC, erlotinib-resistant NSCLC, and in a PDX model. Our results indicated that GNA efficiently overcomes erlotinib resistance in NSCLC in vitro and in vivo, suggesting the potential application of GNA on NSCLC clinical study.

## Results

### GNA inhibits FGFR signaling pathway

**Fig. 1:**
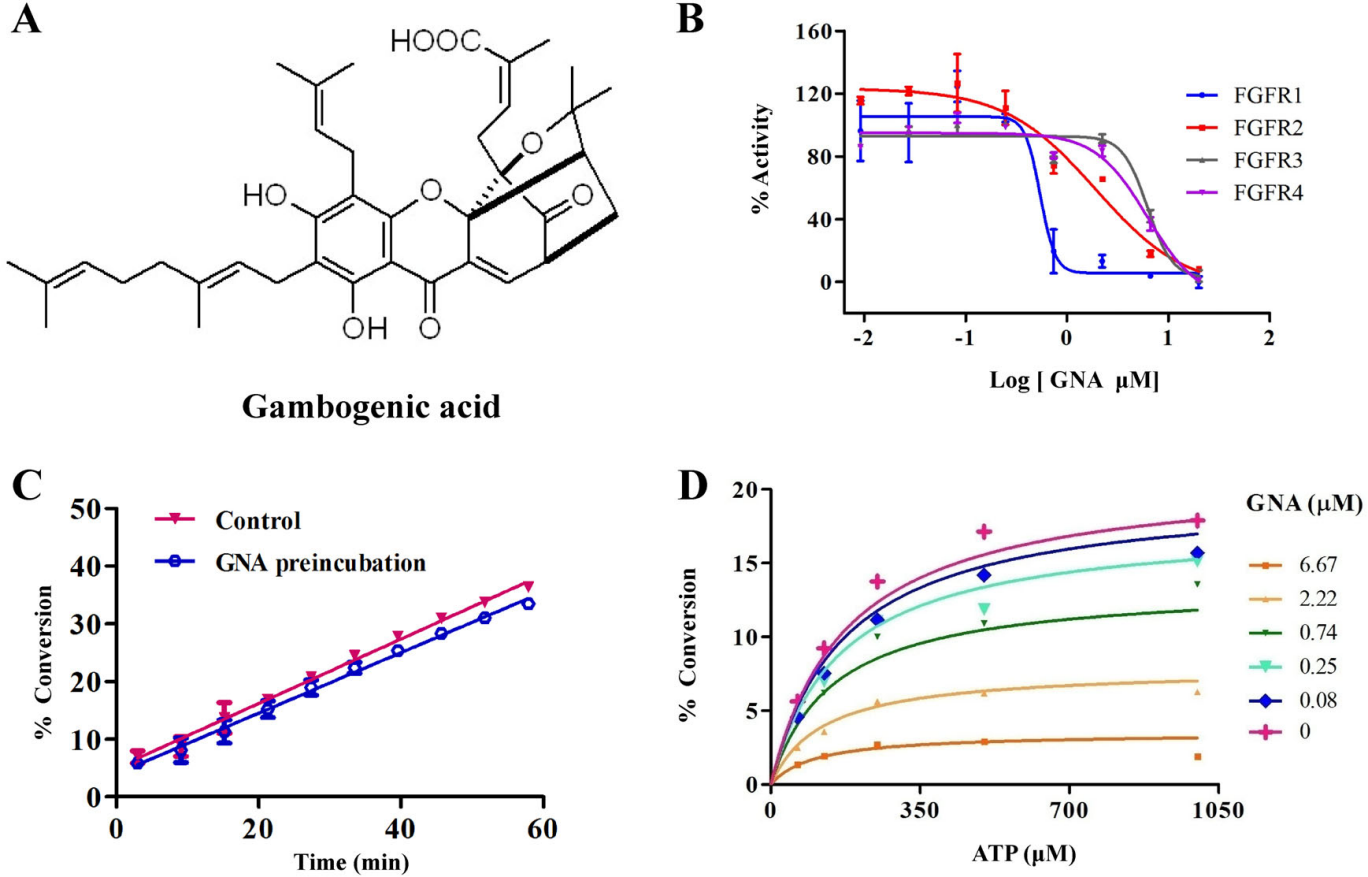
GNA inhibits fibroblast growth factor receptors (FGFRs) and multiple downstream kinases. **a** The chemical structure of GNA. **b** GNA was tested in an FGFR 1-4 kinase assay starting from 10 µm with threefold dilutions. **c** The reversible assay was performed by pre-incubating GNA with FGFR1 for 30 min and diluted in the substrate mixture. The signal was read in real time by a caliper mobility shift assay. **d** The ATP-competitive kinase assay of GNA with FGFR1 was carried out by a caliper mobility shift assay. The conversion data were fitted with GraphPad using a mixed mode inhibition equation

Dysregulation of protein kinase activity is known to be a key factor in tumorigenesis^20^. The receptor tyrosine kinases include EGFR, insulin-like growth factor-1 receptor, vascular endothelial growth factor receptor, FGFR1, FGFR3, and FGFR4, FMS-like tyrosine kinase, c-Met, and c-KIT^21^. The protein kinases of the PI3K/AKT pathway (such as PI3K, AKT, and mTOR) are known to play an important role in cell growth and survival^22^. We then performed a mobility shift assay to evaluate the effect of GNA on FGFR and its downstream kinases (Fig. 1a for GNA structure). GNA inhibited multiple kinases, and the IC_50s_ were mainly in the range of 1.23 μm to 10 μm (Supplementary Figure 1 and Supplementary Table 1). Notably, GNA had a high efficacy for inhibiting FGFR family proteins, especially for FGFR1 and FGFR2 (Fig. 1b). We were curious about whether the binding between GNA and FGFR1 was irreversible and dependent on ATP. The reversibility assay was run through rapid dilution after a pre-incubation with FGFR1 and GNA. There was no significant difference between GNA/FGFR1 and FGFR1 alone, suggesting GNA binding reversibly to FGFR1 with rapid association and dissociation rates (Fig. 1c). Furthermore, an ATP competitive assay was performed to investigate the GNA and FGFR1 binding manner. As shown in Fig. 1d, the alpha fitting value was 0.55, indicating that GNA displays noncompetitive inhibition in both the free enzyme and enzyme-substrate binary complex. Therefore, GNA inhibited FGFR1 in an ATP independent manner. The inhibitory effect of GNA on c-Met and EGFR was evaluated in vitro. A kinase enzyme assay showed that GNA inhibited the kinase activity of EGFR and cMet (Supplementary Figure 2A and 2B). GNA also inhibited mutated EGFR activity on several EGFR mutants, including EGFR (d746-750), EGFR (L858R), EGFR (T790M + L858R), EGFR(d746-750 + T790M), and EGFR (T790M) (Supplementary Figure 2C). In addition, we examined the effect of GNA on the phosphorylation of EGFR, cMet, AKT, and S6K1 (Supplementary Figure 2D−2G). Taken together, GNA inhibited multiple kinases, such as FGFR, EGFR, and the related downstream signaling, indicating its potential to inhibit NSCLC cell growth.

### GNA inhibits cell proliferation on both NSCLC and erlotinib-resistant NSCLC by targeting FGFR signaling pathway

**Fig. 2:**
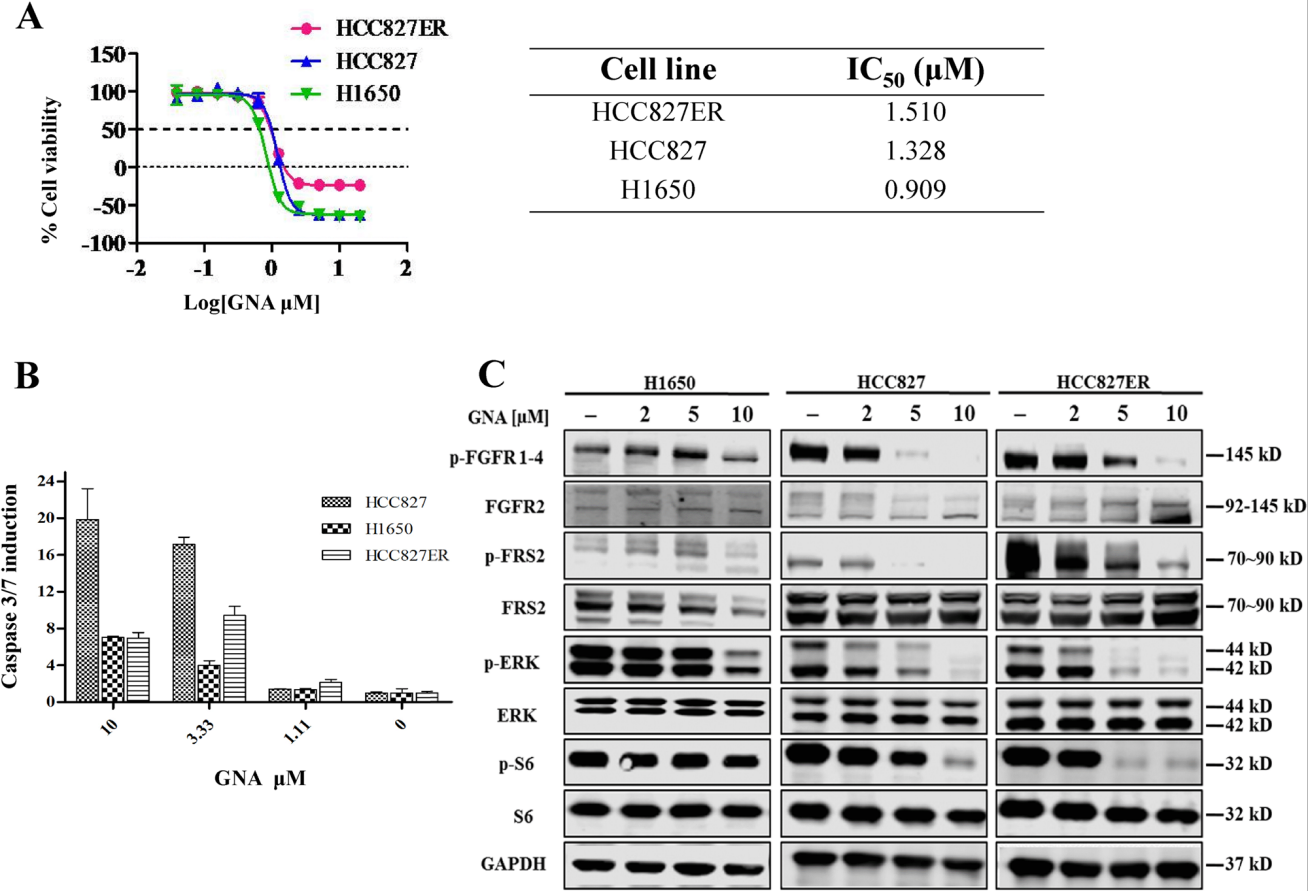
GNA inhibits cell proliferation in both NSCLC and erlotinib-resistant NSCLC by targeting FGFR and the downstream signaling pathways. **a** Cell viability was assessed using the CellTiter-Glo assay. After a 72 h treatment by GNA, CellTiter-Glo reagent was added directly to each well for a 10-min incubation. The plate was then read on a FlexStation 3 microplate luminometer. A dose−response curve was plotted, and the IC_50_ was calculated using GraphPad Prism software. Cell viability was assessed using MTT assay. After a 72 h treatment by GNA, 10 μl of 5 mg/ml 3-(4,5-dimethylthiazol-2-yl)-2,5-diphenyltetrazolium bromide (MTT) solution was added to each well and incubated for 4 h at 37 °C, then the culture medium was removed and 100 μl of DMSO was added to each well. The absorbance was measured at 570 nm on a microplate reader. A dose−response curve was plotted, and the IC_50_ was calculated using GraphPad Prism software. **b** Caspase-3/7 activity induction was evaluated after 6 h of treatment with GNA at 10, 3.33 and 1.11 μM using the Caspase-Glo 3/7 kit from Promega in the HCC827, H1650, and HCC827ER cells, respectively. The bars in the graphs represent the mean fold induction relative to the DMSO control. **c** H1650 cells, HCC827 cells, and HCC827ER cells were treated with GNA at various dosages for 4 h and were then probed with specified antibodies. FGFR and the downstream proteins were analyzed by western blot. **d** The histograms of quantitative analysis about FGFR and the downstream proteins were analyzed by western blot

The effect of GNA on cell proliferation was tested in the NSCLC cell lines using a CellTiter-Glo assay and MTT assay. GNA inhibited the proliferation of HCC827, H1650, and HCC827 erlotinib-resistant (HCC827ER) cells with similar IC_50_ values (Fig. 2a, b). The HCC827ER cells were less sensitive to erlotinib than the HCC827 parental cells as shown by the colony formation and cell proliferation assays (Supplementary Figure 3A and 3B). GNA also induced Caspase-3/7 activation in these cell lines (Fig. 2c), suggesting the apoptotic induction ability of GNA. We then investigated the effect of GNA on FGFR and its downstream signals in these cell lines. As shown in Fig. 2d, GNA efficiently inhibited the phosphorylation of FGFR and its downstream kinases, such as FRS2, ERK, and S6, without interfering with the total protein levels. The quantification of each western blotting was shown in Supplementary Figure 4.

We then carefully analyzed the characteristics of the HCC827ER cell line to gain a better understanding of the drug-resistant mechanism. Supplementary Figure 3C and 3D suggested that the HCC827ER cells contained a higher basal c-Met expression, meanwhile the HCC827ER cells had a higher PARP protein and a higher phosphorylation of c-Met, AKT, ERK, Stat3, and S6, which cannot be attenuated by erlotinib treatment. Moreover, the droplet digital PCR (ddPCR) analysis revealed that there was no L858R and T790M mutations in the EGFR gene of HCC827ER cells, suggesting that the drug resistance is not related to EGFR activity (Supplementary Figure 3E to 3G).

### GNA potentiates the therapeutic efficacy of erlotinib on NSCLC in vitro

**Fig. 3:**
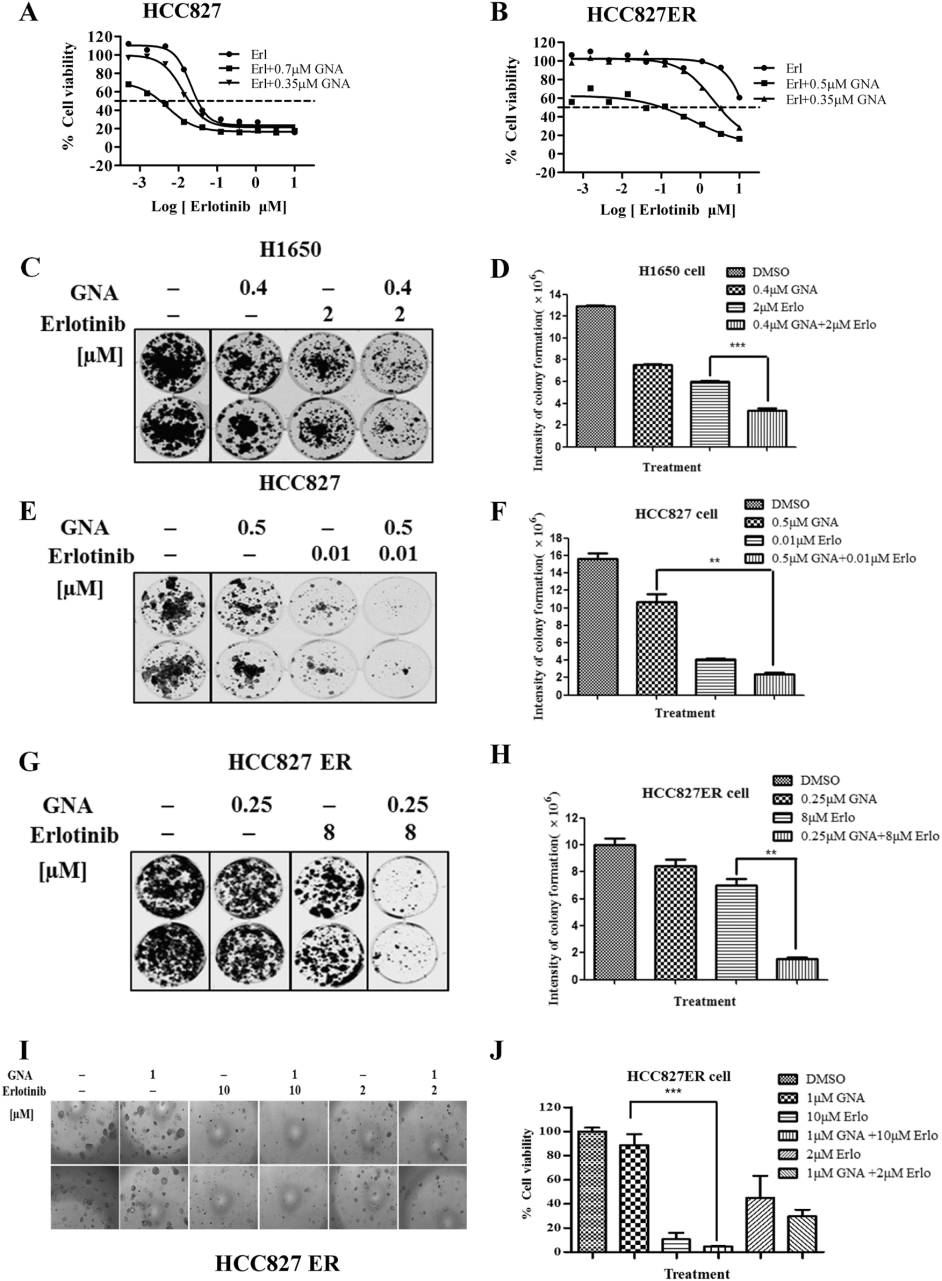
GNA potentiates the therapeutic effect of erlotinib on NSCLC in vitro. **a** HCC827 and **b** HCC827 ER cell lines were treated by titrated erlotinib in the presence of a fixed concentration of GNA for 72 h, and the cell viability was assessed using the CellTiter-Glo assay. **c**−**h** Colony formation assay. **c** H1650, **e** HCC827, and **g** HCC827 ER cell lines were seeded at a certain cell density per well in six-well plates and were incubated overnight, and then, the cells were treated with different concentrations of GNA, erlotinib, or their combination. DMSO was used as the vehicle control. The culture media was removed and replaced with fresh media with or without compounds every 72 h for a total of 14 days. The histograms of quantitative analysis about colony formation assay. **d** H1650, **f** HCC827 and **h** HCC827ER cell lines. **i** Soft-agar colony formation assay. HCC827ER cells were plated in soft agar in a 96-well plate and were incubated overnight. After that, the cells were treated with GNA, erlotinib, or a combination of the two for 10 days. **j** CellTiter blue reagent was added and incubated overnight. The fluorescent signal was read by a FlexStation plate reader. The data are presented as the means ± SD and were analyzed by a one-way ANOVA, *P < 0.05, **P < 0.01, ***P < 0.001

Since GNA inhibited NSCLC cell proliferation independent of the EGFR mutant status, we speculated whether GNA had a synergetic effect with erlotinib in NSCLC in vitro. First, we performed a panel screen to calculate the combination index (CI) of GNA and erlotinib in multiple NSCLC cell lines (Supplementary Table 2). The CI values at ED75, ED90, and ED95 were less than 1 for 10 of the 21 NSCLC cell lines highlighted in gray. Specifically, the CellTiter-Glo assay showed that GNA sensitized the HCC827 and HCC827ER cells to erlotinib treatment (Fig. 3a, b). The clonogenic assays indicated that GNA and erlotinib exhibited a synergetic effect on colony formation in H1650 and HCC827 cells (Fig. 3c, f). Interestingly, a combination of 0.25 μm GNA and 8 μm erlotinib completely blocked colony formation in HCC827 ER cells, which was only slightly inhibited by either agent alone (Fig. 3g, h). GNA also potentiated the inhibitory effect of erlotinib on HCC827ER cells in the 3D agar colony formation analysis (Fig. 3i, j).

**Fig. 4:**
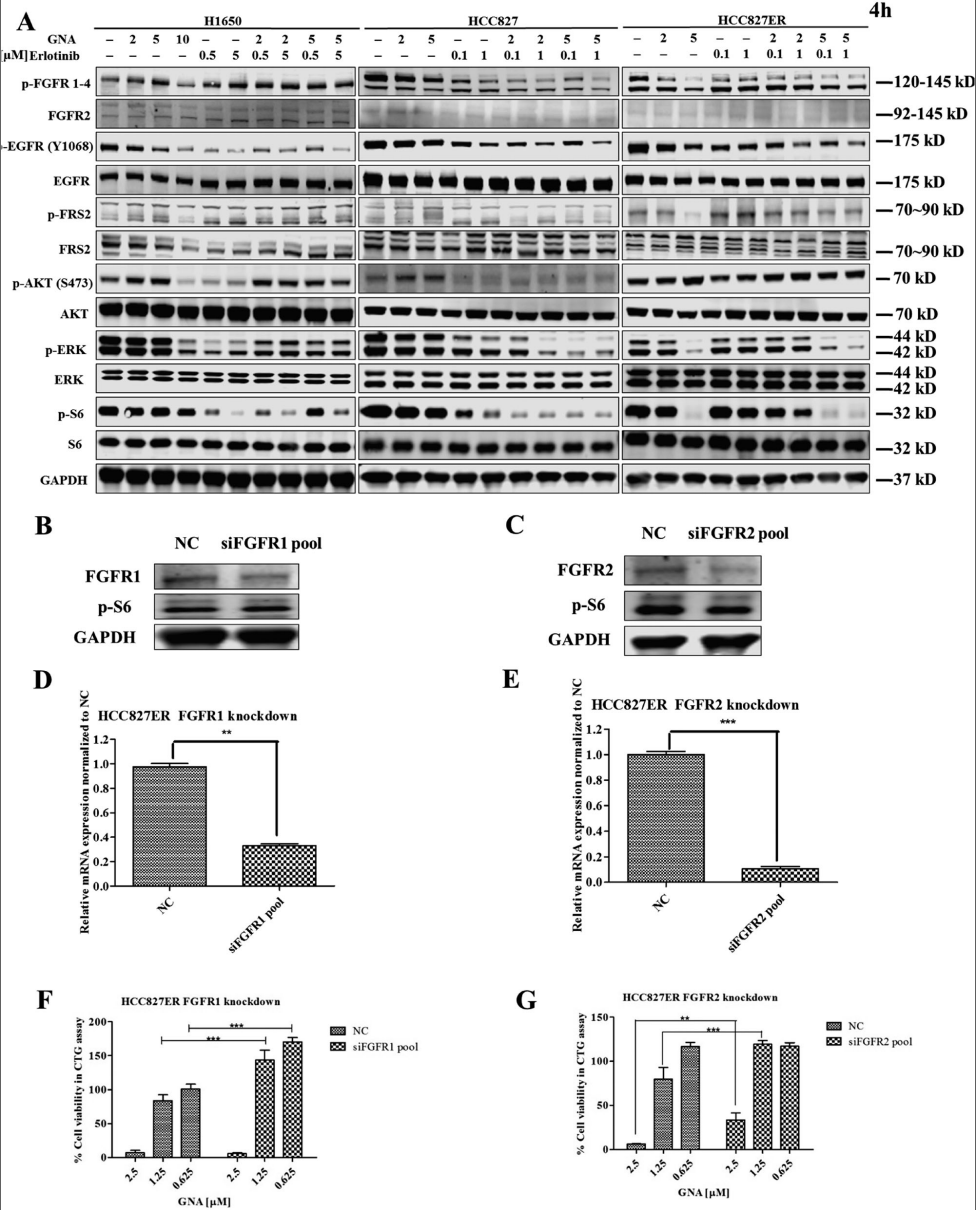
GNA enhances the anti-tumor activity of erlotinib through FGFR in vitro. **a** H1650 cells, HCC827 cells, and HCC827ER cells were treated with various concentrations of GNA, erlotinib, or their combination for 24 h and were then probed with specified antibodies. **b**−**e** FGFR and the downstream proteins were analyzed by a western blot. HCC827ER cells were transfected with FGFR1 and FGFR2 siRNA for 48 h, then the medium was replaced and the cells were further incubated for 24 h. After incubation, the protein expression of FGFR1 (**b**) and FGFR2 (**c**) were analyzed by western blot, the mRNA expression of FGFR1 (**d**) and FGFR2 (**e**) were analyzed by real-time PCR. (**f**, **g**) FGFR1 or FGFR2 knockdown HCC827ER cells were treated by GNA for 72 h, and the cell viability was assessed using the CellTiter-Glo assay. The data are presented as the means ± SD and were analyzed by a one-way ANOVA, **P < 0.01, ***P < 0.001

We then performed the mechanism study on the combination of GNA and erlotinib on H1650, HCC827, and HCC827ER cell lines. As shown in Fig. 4a, the phosphorylation and the total protein levels of FGFR, EGFR, and the downstream signals, including AKT, ERK, and S6, were analyzed with various doses of GNA, erlotinib, or the combination treatment. The combination treatment caused a reduction in several proteins, such as FRS2, ERK, and S6. Specifically, we carefully compared the effects of the different treatments on the EGFR signaling pathway in the HCC827 and HCC827ER cell lines and found that the hyperactivation of c-Met, ERK, S6, and Flt3 was attenuated by the combined treatment (Supplementary Figure 5A and 5B).

To examine whether the FGFRs play essential role in the HCC827ER cells, we performed FGFRs knockdown by using siRNA pools of FGFR1 and FGFR2 and investigated the effects against GNA treatment. As shown in Fig. 4b, e, the FGFR1 and FGFR2 were efficiently knocked down in HCC827ER cells both in mRNA and in protein level by transfecting siRNA pools, respectively. We then evaluated the GNA sensitivity of the HCC827ER in the FGFR1 and FGFR2 knockdown cells by Cell Titer-Glo cell viability assay. Figures 4f, g indicated that knockdown of FGFR1 and FGFR2 could decrease the sensitivity upon GNA treatment in HCC827ER cell, suggesting FGFRs played an essential role in mediating the GNA-induced cell death.

### GNA reverts erlotinib resistance in a xenograft and PDX model

It is important to understand the effect of GNA on NSCLC in an animal model, especially in erlotinib-resistant cell lines. To evaluate the preclinical efficacy, we established a xenograft model using HCC827 and erlotinib-resistant HCC827ER cell lines. We first examined the effect of erlotinib on HCC827 with or without GNA administration. GA, a compound undergoing a clinical trial in China, was also used as reference^23^. GNA, erlotinib, and their combination retarded tumor growth, at day 22 compared to the vehicle group (Supplementary Figure 6A). The mice body weight was not affected by GNA, erlotinib, or their combination (Supplementary Figure 6B). The synergetic effect of GNA and erlotinib calculated as *Q* value is 1.17.

**Fig. 5:**
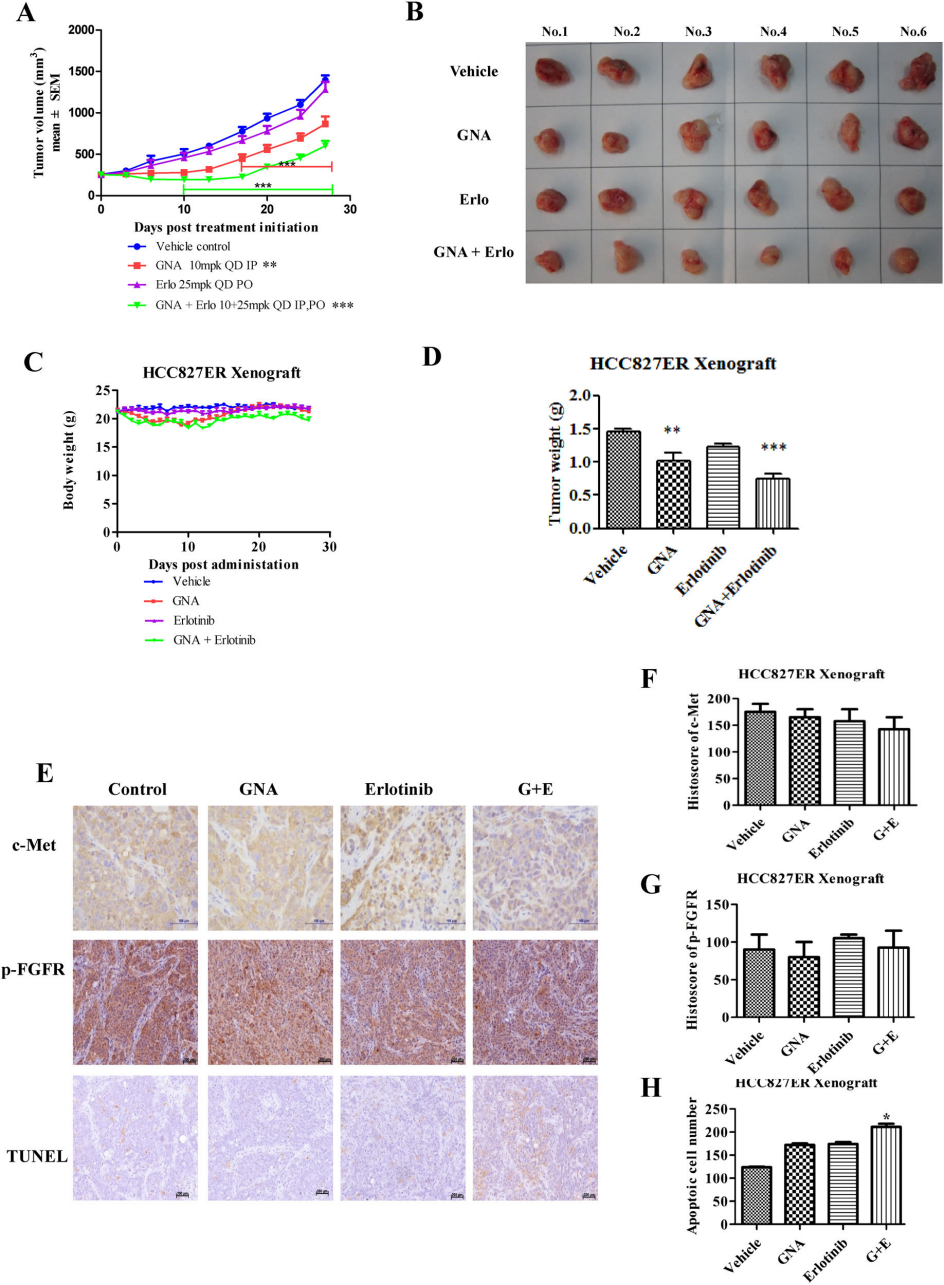
GNA increases the anti-tumor activity of erlotinib in erlotinib-resistant xenograft model. **a** HCC827ER cells were implanted subcutaneously into the right flank of nude mice. When the tumors reached 150−250 mm^3^, the tumor-bearing mice were then treated with vehicle, GNA (i.p.,10 mg/kg), erlotinib (p.o., 25 mg/kg), or their combination once daily for 27 days. The tumor volume was calculated by determining the length and width of the tumor as measured using Vernier calipers. The data are presented as the means ± SEM (*n* = 6) and were analyzed by a one-way ANOVA, *P < 0.05, **P < 0.01, ***P < 0.001. **b** Images of the representative tumors from (**a**). **c** The body weights of the mice from (**a**). **d** The tumor weight ratio of the mice from (**a**). **e**−**g** Representative immunohistochemical image analyses for c-Met (**e**, **f**) and apoptotic cells by a TUNEL assay (**e**, **g**) in the xenograft mouse model of the HCC827ER tumors after vehicle, GNA, erlotinib, and combination treatment. The data are presented as the Histoscore and the error bars are the SEM. Statistically significant differences with *P* < 0.05 were considered significant compared with the vehicle control, *P < 0.05

For the HCC827ER xenografts, tumor growth was not inhibited in the erlotinib-treated group (Fig. 5a, b). GNA alone or GNA and erlotinib in combination managed to suppress the tumor growth in the HCC827ER xenograft. In addition, the combination of GNA and erlotinib showed a significant synergistic effect from day 10 (*Q* = 1.29) after drug administration. Meanwhile, the body weight did not show any difference between each group, and the tumor weight indicated a significant effect in the combination treatment group (Fig. 5c, d). These results confirmed that GNA sensitized the tumor to erlotinib even in erlotinib-resistant cells, suggesting the potential of GNA in clinical application. Since the hyperactivation of c-Met was observed in the HCC827ER cells, we then evaluated c-Met by IHC in the xenografts. As shown in Fig. 5e, h, the combined treatment decreased c-Met in the xenografts compared to the other groups. The TUNEL assay indicated that the combined treatment efficiently induced apoptosis (*P* < 0.05). In brief summary, these results demonstrated that GNA and erlotinib worked synergistically in erlotinib-resistant xenograft in vivo.

**Fig. 6:**
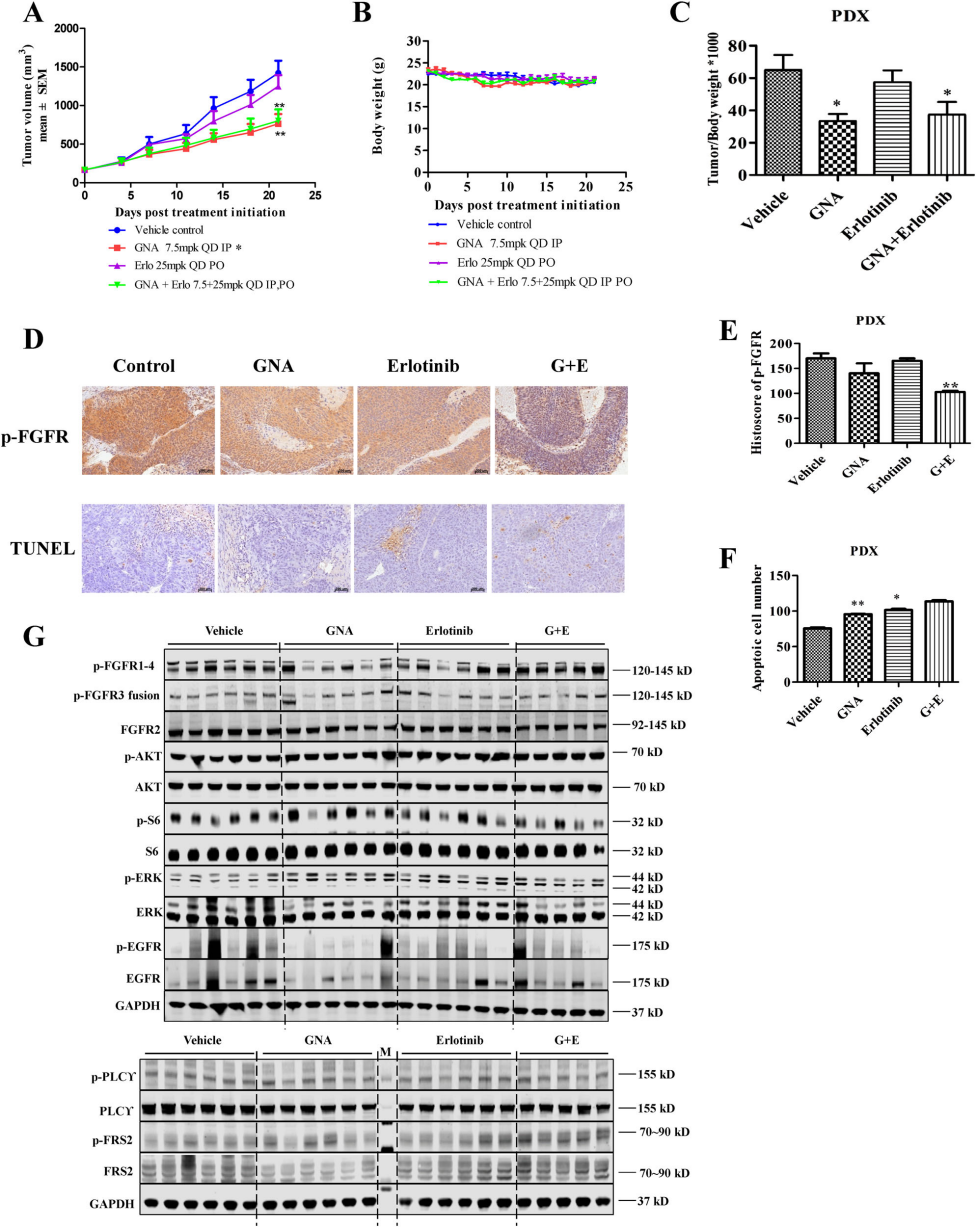
GNA increases the anti-tumor activity of erlotinib in FGFR-expressing PDX xenograft model. **a** The fragments from the donor mice that were implanted from patient samples were implanted subcutaneously into the right flank of nude mice. When the tumors reached 150–250 mm^3^, the tumor-bearing mice were then treated with vehicle, GNA (i.p., 7.5 mg/kg), erlotinib (p.o., 25 mg/kg), or their combination once daily for 22 days. The tumor volume was calculated by determining the length and width of the tumor as measured using Vernier calipers. The data are presented as the means ± SEM (*n* = 6) and were analyzed by a one-way ANOVA, *P < 0.05, **P < 0.01, ***P < 0.001. **b** The body weight of the mice from (**a**). **c** The tumor/body weight ratio of the mice from (**a**). **d**−**f** Representative immunohistochemical image analyses for p-FGFR (**d**, **e**) and apoptotic cells by a TUNEL assay (**d**, **f**) in the xenograft mouse model of the PDX tumors after vehicle, GNA, erlotinib, and combination treatment. The data are presented as the Histoscore and the error bars are the SEM. Statistically significant differences with *P* < 0.05 were considered significant compared with the vehicle control, *P < 0.05, and **P < 0.01. **g** Inhibition of FGFR phosphorylation in NSCLC PDX by GNA. After treatment with GNA, erlotinib, and its combination for about 3 weeks, the tumors were collected and processed. The protein samples were then probed with specified antibodies

It was of interest to further explore the capability of GNA or the GNA and erlotinib combination in the PDX model. First, we characterized the mutant genes in the PDX model using RNA-seq. As shown in Supplementary Table 3, the hyperactivation of FGFR family proteins and some survival factors, such as AKT, ERK, and PI3K, was identified in the tumor. Since EGFR/cMet expression was low (FPKM < 30) and FGFR3-TACC3 fusion was high FPKM (>250), we speculated that GNA mainly targeted the FGFR signaling pathway, such as the FGFR3 fusion proteins in the PDX model. As expected, erlotinib administration did not suppress tumor growth in the PDX model. On the contrary, GNA administration attenuated the tumor growth (Fig. 6a). The body weight of all the animals was not affected in each group nor was the tumor/body weight ratio (Fig. 6b, c). We then performed IHC staining to examine the effect of the drug administration on the phosphorylation of FGFR. As shown in Fig. 6d, e, the suppression of p-FGFR was observed in the GNA or GNA and erlotinib combined treatment, although the GNA alone did not show a significant difference due to the sample quantity limitation. The TUNEL assay further suggested that GNA efficiently induced apoptosis (*P* < 0.05), but erlotinib did not interfere with the PDX growth in vivo (Fig. 6d, f). Lastly, we carefully analyzed the biochemical changes, focusing on the FGFR and EGFR signaling pathways, in the PDX model by a western blot. Figure 6g shows differences in the group even with the same treatment. Nevertheless, the suppression of some of kinases, such as p-FGFR1-4, p-S6, and p-EGFR, was observed.

Taken together, in the present study, we systematically analyzed the mechanism of action of GNA in NSCLC and in an erlotinib-resistant HCC827 cell line. We performed kinase activity assays, cell-based assays, and an in vivo evaluation of GNA in multiple xenograft models. Our data provided evidence that GNA exhibited strong anticancer activity against erlotinib NSCLC, and it should be further developed as a lead compound in preclinical studies.

## Discussion

Caged xanthones, a special natural compound family, have gained great attention because of their diverse bioactivities, especially in anticancer^24^. For instance, GA, the most well-studied representative, is reported to target several important signaling pathways, including apoptosis, autophagy, metastasis, and angiogenesis^25^. Therefore, many proteins interact with GA, and these interactions explain the pharmacological mechanisms of GA^26^. Our previous study found that GNA had a profound effect on inhibiting NSCLC cell proliferation^19^. In this study, we further examined the effects of GNA on the key signaling pathways, such as FGFR and EGFR, in NSCLC cell lines. Interestingly, our data showed that GNA inhibited multiple kinases, including receptor kinases, such as EGFR, cMet, and FGFR. NSCLC cancer cells are characterized by the aberrant activation of EGFR, ALK, or cMet^27^. In clinical practice, drug resistance develops after several months of treatment with the EGFR inhibitor erlotinib, and the reason behind it is not clear. Some studies link the resistance to c-Met overexpression in NSCLCs^28,29^, which is also confirmed in the current study. Thus, a treatment targeting both receptors may delay the development of resistance in NSCLC patients after erlotinib treatment.

EGFR and cMet physically bind together, and the interaction leads to cMet hyperactivation followed by drug resistance^30–32^. In our study, we found that the inhibition of cMet potentiated the inhibitory effect of erlotinib. First, we showed that GNA potentiated the inhibitory effect of erlotinib on lung cancer cell proliferation, colony formation, tumor growth, and EGFR phosphorylation, which correlated with the inhibition of cMet activity. Second, using the cMet/ALK- specific inhibitor crizotinib, we confirmed that the inhibition of cMet/ALK enhanced the inhibitory effect of erlotinib on cell proliferation and EGFR activity. To our knowledge, this is the first study to show that cMet influences EGFR phosphorylation. It would be interesting to explore this finding further to understand the detailed regulation mechanism between EGFR and cMet.

From the CDX and PDX in vivo models, we found that only when there was a higher expression of EGFR did the combination of GNA and erlotinib enhance the anti-tumor effect of erlotinib. In the PDX model, erlotinib did not show an anti-tumor effect, and the combination of GNA and erlotinib showed the same efficacy as with GNA alone due to the low EGFR expression. These data also suggest that it is important to classify the genomic information before applying targeted drugs against NSCLC. In other words, it will be valuable to examine the effect of GNA with other chemotherapeutics using both in vitro and in vivo models in future studies.

In conclusion, our study showed that GNA inhibits lung cancer cell proliferation and tumor growth as a multiple kinase inhibitor, which is probably through the inhibition of cMet, EGFR, and other kinases. Our study suggests that the inhibition of cMet, FGFR, or non-EGFR receptor tyrosine kinases (RTK) may potentiate the inhibitory effect of erlotinib in NSCLC with a higher EGFR expression, especially for acquired erlotinib-resistant lung cancer.

## Materials and methods

### Cell culture and chemicals

All the lung cancer cell lines were obtained from ATCC and were cultured in the supplier’s recommended media supplemented with 10% FBS. All the cells were grown in the presence of penicillin−streptomycin at 37 °C and 5% CO_2_. Erlotinib (S7786) and critizonib (S1068) were purchased from Selleck, Inc., China. GNA and GA was ordered from PI & PI Technology, Inc., China. All consumables and regular reagents for the experiments were purchased from Sigma-Aldrich.

### **Generation of erlotinib-resistant HCC827 lung cancer cell line**

To generate resistant cells, we cultured the HCC827 cells in growth medium in the presence of vehicle (0.1% DMSO) or gradually increasing concentrations of erlotinib from 0.002 to 0.2 µm over 3–4 months and with 30 passages to result in the resistant cell line that was designated HCC827-ER.

### Droplet digital PCR (ddPCR) analysis

The DNAeasy Kit (Qiagen) was used according to the manufacturer’s instructions to extract genomic DNA from the HCC827 and HCC827 ER cell lines. ddPCR was then performed to detect the EGFR gene mutation statuses of the cell samples as directed by the supplier’s instructions using a QX200 AutoDG Droplet Digital PCR system (Bio-Rad Laboratories, Inc.). The data were analyzed using QuantaSoft software (Bio-Rad Laboratories, Inc.).

### Caspase-Glo 3/7 assay

Caspase-3/7 activities were examined using the Caspase-Glo 3/7 kits according to the manufacturer’s protocol (Promega, Madison, WI). The assay was run as described in the previous paper^19^. Finally, the signal was read using a FlexStation3 microplate reader (Molecular Devices, USA).

### **Dose−response cell viability assay and 2D and 3D** c**lonogenic assays**

Unless indicated otherwise, all the cell viability assays were performed using the Cell Titer-Glo assay (Promega). The cells were counted and seeded in 96-well plates. The next day, the compounds were added, and 3–5 days later cell viability was assessed. The IC_50_ determination was performed using GraphPad Prism. The Chou−Talalay drug combination (CI) indices were calculated by CompuSyn software. A combination index <1 is considered synergistic.

For the 2D colony formation assay, 3000–5000 cells were seeded on six-well plates and were treated with drug or vehicle control for 2–3 weeks until clear colonies formed. The colonies were fixed with 20% PREFER CONCENTRATE (Ref 411, ANATECH LTD.) and stained with 0.1% crystal violet. Images were captured, and the number of colonies in each well was counted using an Odyssey Imaging system (LICOR, USA).

For the 3D clonogenic assay, soft-agar colony formation was performed using previously described methods^19^. Briefly, the cells were plated in soft agar (Takara, Japan) at a density of 2000–3000 cells in a 96-well plate and were treated with drug and vehicle control for 7–10 days until the colonies formed. The colonies were stained with Alamar blue in incubator overnight. The fluorescence intensity (excitation 560 nm, emission 590 nm) was measured in a FlexStation plate reader.

### Western blotting

The cells were lysed with ice-cold 1× RIPA lysis buffer (CST#9806) supplemented with a complete mini protease inhibitor cocktail (Roche Applied Science, Indianapolis, IN). The lysates were sonicated, centrifuged, and boiled with reducing sample buffer. The protein samples were separated by SDS-polyacrylamide gel electrophoresis on 4–12% gradient gels (Invitrogen) and were transferred onto nitrocellulose membranes. The following primary antibodies were used in this study: p-FGFR1-4 (R&D#AF3285); FGFR2 (Abcam#ab109372); p-FRS2(R&D#5126); FRS2 (R&D#MAB4069); p-Met (CST#3077); Met (CST#8198); p-S6 (CST#4856); S6 (CST#2217); p-EGFR (CST#2236); EGFR (CST#2236); p44/42 (CST#9107); p-p44/42 (CST#4370); AKT (CST#9272); p-AKT (CST#9271); p-ALK(CST#3341); ALK(CST#3333); STAT3 (CST#9139); and p-STAT3 (CST#9131). Unless indicated otherwise, all antibodies were used at a 1:1000 dilution, except for GAPDH, which was used at a 1:5000 dilution. The protein bands were detected using an Odyssey Imaging system (LICOR, USA).

### **Kinase activity assay by a microfluidic mobility shift enzyme assay**

The mobility shift enzyme assay was performed as previously described^19^. Briefly, the test compounds were diluted and mixed with substrate, and then, the enzyme was added to initiate the reaction. After a period of incubation at room temperature, the reaction was stopped by a stop solution. Finally, the plate was put on a Caliper Labchip 2000 system, and a droplet of the reaction mixture was applied for electrophoretic separation in the chips of the machine. The enzyme conversion data were then readout for analysis.

### GNA reversible assay and ATP competitive inhibition assay

To determine GNA reversibility, 400 nm of FGFR1 and 5 μm of GNA or 2% DMSO were preincubated for 30 min respectively. Then the mixture was diluted into substrates with 100-fold dilution. The assay was initiated and the signal was detected in real time through caliper mobility shift assay.

To test the relationship between GNA and ATP, the concentration of the peptide substrate was constant, while the concentrations of ATP were set at 1000, 500, 250, 125, and 62.5 μm. The global competitive inhibition fit for the compounds was executed based on the mixed model inhibition equation in the Graphpad Prism software.

### In vitro EGFR/c-Met/AKT/S6K1 phosphorylation assay

For EGFR pan-tyrosine phosphorylation, A431 cells were seeded in 96-well plates with DMEM containing 10% FBS for 24 h, followed by serum starvation (0.1% FBS) for 24 h. The cells were incubated with GNA for 60 min before stimulation for 20 min with 100 ng/ml recombinant EGF (R&D Systems). The cells were lysed in 1× cell lysis buffer (CST#9803) plus 1 mm phenyl-methylsulfonyl fluoride (PMSF). EGFR phosphorylation was measured by a sandwich ELISA (CST#7911S) according to the manufacturer’s instructions.

For c-Met pan-tyrosine phosphorylation, HCC827 cells were seeded in 96-well plates with RPMI 1640 containing 10% FBS for 24 h and were then incubated with GNA for 3 h. The cells were lysed in 1× cell lysis buffer (CST#9803) plus 1 mm PMSF. c-Met phosphorylation was measured by a sandwich ELISA (CST#7333S) according to the manufacturer’s instructions.

For the inhibition of PI3K, AKT phosphorylation was examined. Raji cells were seeded in 96-well plates with RPMI 1640 containing 10% FBS for 2 h and were then incubated with GNA for 2 h before stimulation for 30 min with 0.5 μg/ml α-IgM. The cells were centrifuged and lysed in 1× cell lysis buffer (CST#9803) plus 1 mm of PMSF. AKT phosphorylation was measured by a sandwich ELISA (CST#7252S) according to the manufacturer’s instructions.

For the evaluation of the inhibition of mTOR, S6K1 phosphorylation was examined in AlphaLISA assays. MCF-7 cells were seeded in 384-well plates with DMEM containing 10% FBS for 24 h. On the second day, the culture medium was replaced with fresh medium, the diluted GNA was added and the plate was incubated for 2 h. Then, the cells were lysed with the cell lysis buffer. S6K1 phosphorylation was measured by an AlphaLISA SureFire assay kit (PerkinElmer#ALSU-PP70-A10K) according to the manufacturer’s instructions.

### Transfection of small interfering RNA (siRNA)

siRNA for FGFR1 and FGFR2 and the control siRNA (non-targeting control, NC) were purchased from GenePharma (Shanghai, China). Transfection of each siRNA (40 nm) was performed by using Lipofectamine RNAi-MAX (Invitrogen, USA). After 48 h of transfection, the medium was replaced and the cells were further incubated for 24 h. After incubation, the cells were counted, cell lysates were obtained for mRNA quantification and protein analysis of FGFR1 and FGFR2 respectively, and cell viability were also assessed by CellTiter-Glo assay.

### Real-time qPCR analysis

Total RNA was isolated from erlotinib-resistant HCC827 cell line using the RNeasy plus mini kit according to the manufacturer’s protocol (Qiagen, USA). Then, first-strand cDNA was reverse-transcribed from 2 μg total RNA using high-capacity cDNA reverse transcription kit (Thermo, USA), and amplified by Power SYBR Green PCR master mix kit (Thermo, USA). A master mix was prepared for each PCR reaction, which included Power SYBR Green PCR master mix, forward primer, reverse primer, and 10 ng of template cDNA. PCR conditions were 10 min at 95 °C, followed by 40 cycles at 95 °C for 15 s and 60 °C for 1 min. The forward and reverse primer sequences for FGFR1 were 5′- GTG GCT GGG GTT GTA GCA GTA-3′ and 5′-ACG CAG GAT GGT CCC TTG TAT-3′. The forward and reverse primer sequences for FGFR2 were 5′-TGG CTG GCT TAT CCA TTC TGT-3′ and 5′-GTC TGG TCC TTC GGG GTG TTA-3′.

### CDX and PDX in vivo model

Female Balb/c nude mice between the ages of 6 and 8 weeks, with a body weight of approximately 18–22 g, were ordered from the vendor and were housed in specific pathogen-free (SPF) conditions at ChemPartner. The animals were held for a minimum of 3 days for acclimation prior to the beginning of study.

The animals were provided pelleted food and water ad libitum and were kept in a room conditioned at 20−25 °C, with 40−70% relative humidity, and with light 7:00 am to 7:00 pm according to the AAALAC guide. The body weights and health statuses were monitored twice a week throughout the study period, and the dose was adjusted per body weight. The body weight data are graphically represented as the mean body weight ± standard error of the mean (SEM). The body weights and tumor areas (length × width) were recorded two or three times per week (depends on the growth speed of tumor). Tumor length and width were measured by using digimatic calipers throughout the study period, and tumor volume was calculated based on the following formula: tumor volume = (length × width^2^)/2. The efficacy data are graphically represented as the mean tumor volume ± SEM.

The human cell-derived tumor xenografts (CDX) were established by s.c. injecting 0.2 ml of the tumor cell suspension (1×10^7^ cells) mixed 1:1 with Matrigel (Becton Dickinson). For the PDX model with a synonymous SNV Kras mutation, FGFR fusion and a low expressing EGFR L858R mutation, the PDX from the donor mice that reached approximately 500−1000 mm^3^ were aseptically excised and dissected into fragments of approximately 20 mm^3^, and the fragments from the same passage were implanted subcutaneously via a Trocar needle into female nude mice on the same day.

Once all the CDX and PDX tumors reached a size of approximately 70−260 mm^3^, the mice were randomized into groups of six and were dosed with vehicle, GNA, erlotinib, and their combination. Erlotinib were prepared in a 6% Captisol (Sigma#90990C) in deionized water. GNA/GA was dissolved in saline containing 0.05% DMSO/Tween 80 and then treated by ultrasound. The animals were given GNA (10 mg/kg or 7.5 mg/kg) or GA (5 mg/kg) once (q.d.) by i.p. and erlotinib (25 mg/kg) or vehicle control once (q.d.) by oral gavage. The duration of each study was determined by the tumor growth characteristics, with studies ending once the tumors reached over 0.8 cm^3^. Tumor volume and the percentage of tumor growth inhibition were calculated as described above and a combination analysis was carried out using a *Q* value^33^ (a *Q* value of more than 1.15 was considered a significant synergism).

### **Immunohistochemistry**

c-Met, p-EGFR, p-FGFR, Ki67, and p-FRS2 were evaluated by an IHC analysis with formalin-fixed paraffin-embedded slides sectioned at 4 μm. The following antibodies were used: cMet (CST, #8198); p-FGFR1-4 (R&D#AF3285); p-EGFR (CST#2236); Ki-67(CST#9027); and p-FRS2 (R&D#5126). An in situ cell death detection assay kit (Roche, cat#12156792910) was also used to evaluate the apoptosis as directed by the supplier’s instructions. Slides review method: two pathologists reviewed and scored the IHC-stained slides. The positive intensity (3+ strong, 2+ moderate, 1+ weak and 0 negative) and percentage (0~100%) were marked, and then, the histoscore was calculated using the following formula: Histoscore = (% weak [1+] × 1) + (% moderate [2+] × 2) + (% strong [3+] × 3).

### Statistical analysis

GraphPad Prism software (GraphPad Software) was used for the statistical analyses. Student 2-tailed *t*test was used for comparison between two different groups. ANOVA analysis was used to compare the differences for multiple comparisons. A *P* value of <0.05 was considered statistically significant and indicated as following: *P < 0.05; **P < 0.01; ***P < 0.001.

## Electronic supplementary material

Supplementary Figure 1

Supplementary Figure 2

Supplementary Figure 3

Supplementary Figure 4

Supplementary Figure 5

Supplementary Figure 6

Supplementary Table 1

Supplementary Table 2

Supplementary Table 3
