# Supplementary figures and images for: Gambogenic acid inhibits fibroblast growth factor receptor signaling pathway in erlotinib-resistant non-small-cell lung cancer and suppresses patient-derived xenograft growth
Source: Cell Death Dis. 2018 Feb 15;9(3):262. doi: 10.1038/s41419-018-0314-6 (PMC5833807; doi:10.1038/s41419-018-0314-6)

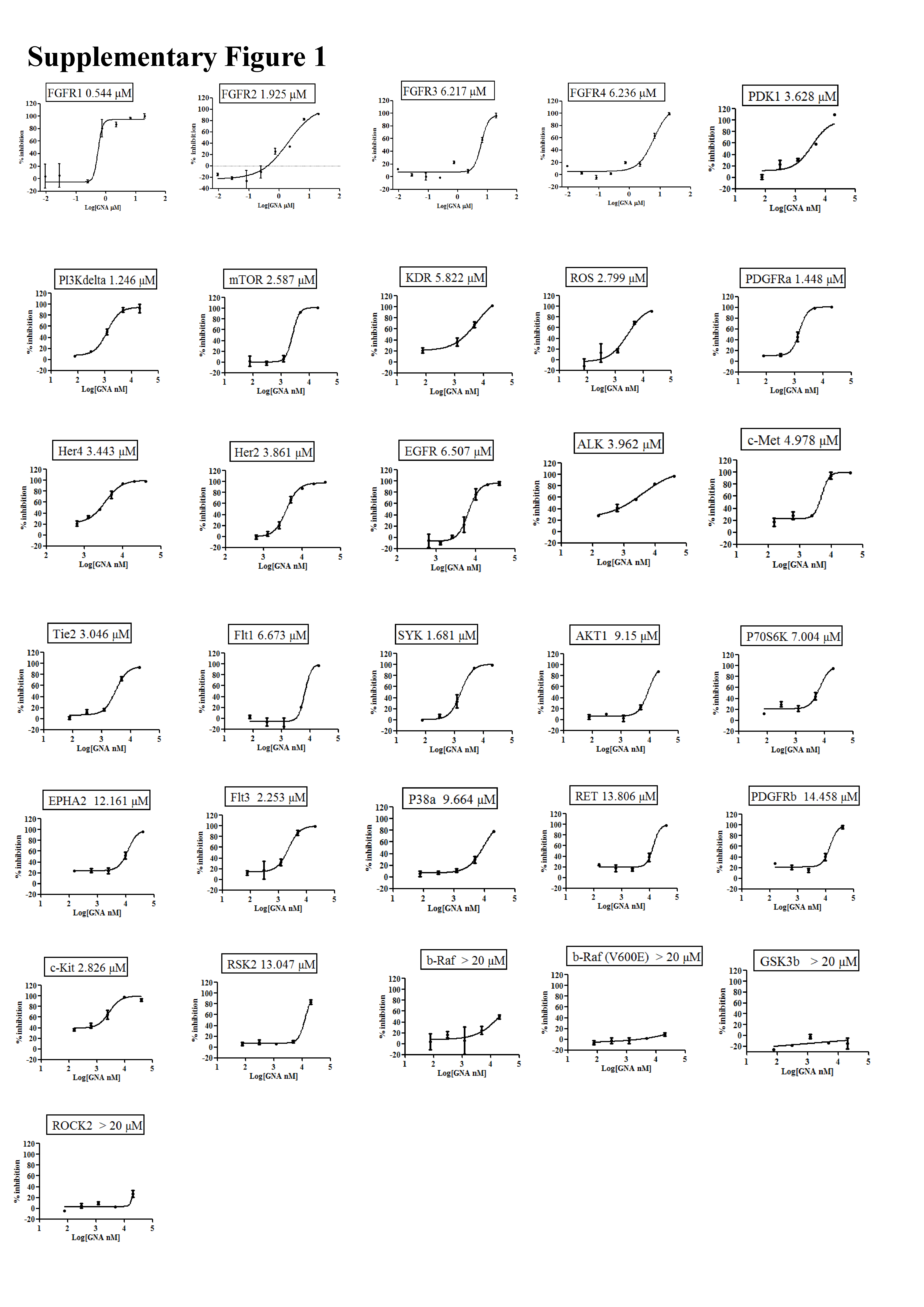

Supplement: Supplementary file 1 — Supplementary Figure 1 [file 41419_2018_314_MOESM1_ESM.tif]

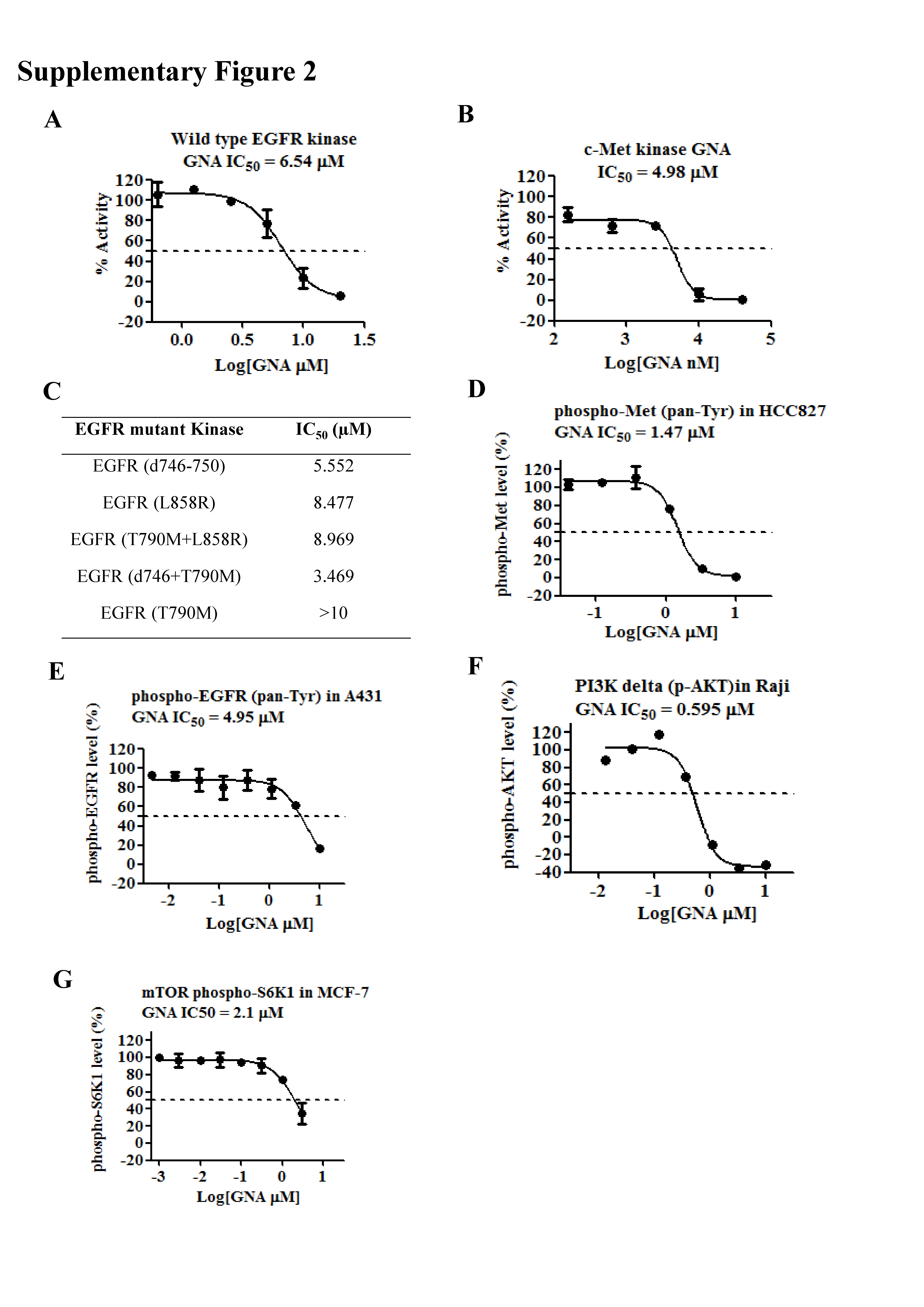

Supplement: Supplementary file 2 — Supplementary Figure 2 [file 41419_2018_314_MOESM2_ESM.tif]

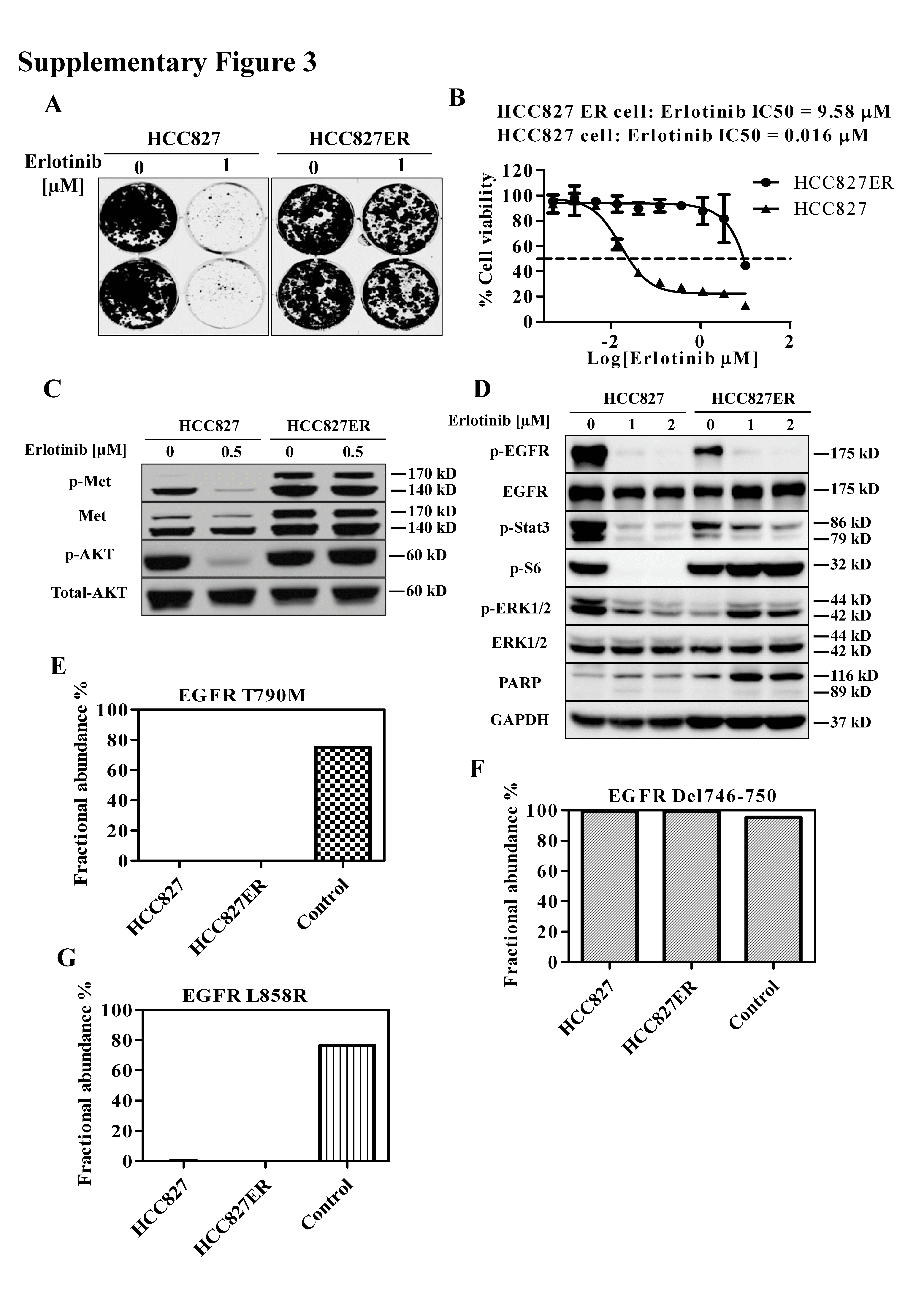

Supplement: Supplementary file 3 — Supplementary Figure 3 [file 41419_2018_314_MOESM3_ESM.tif]

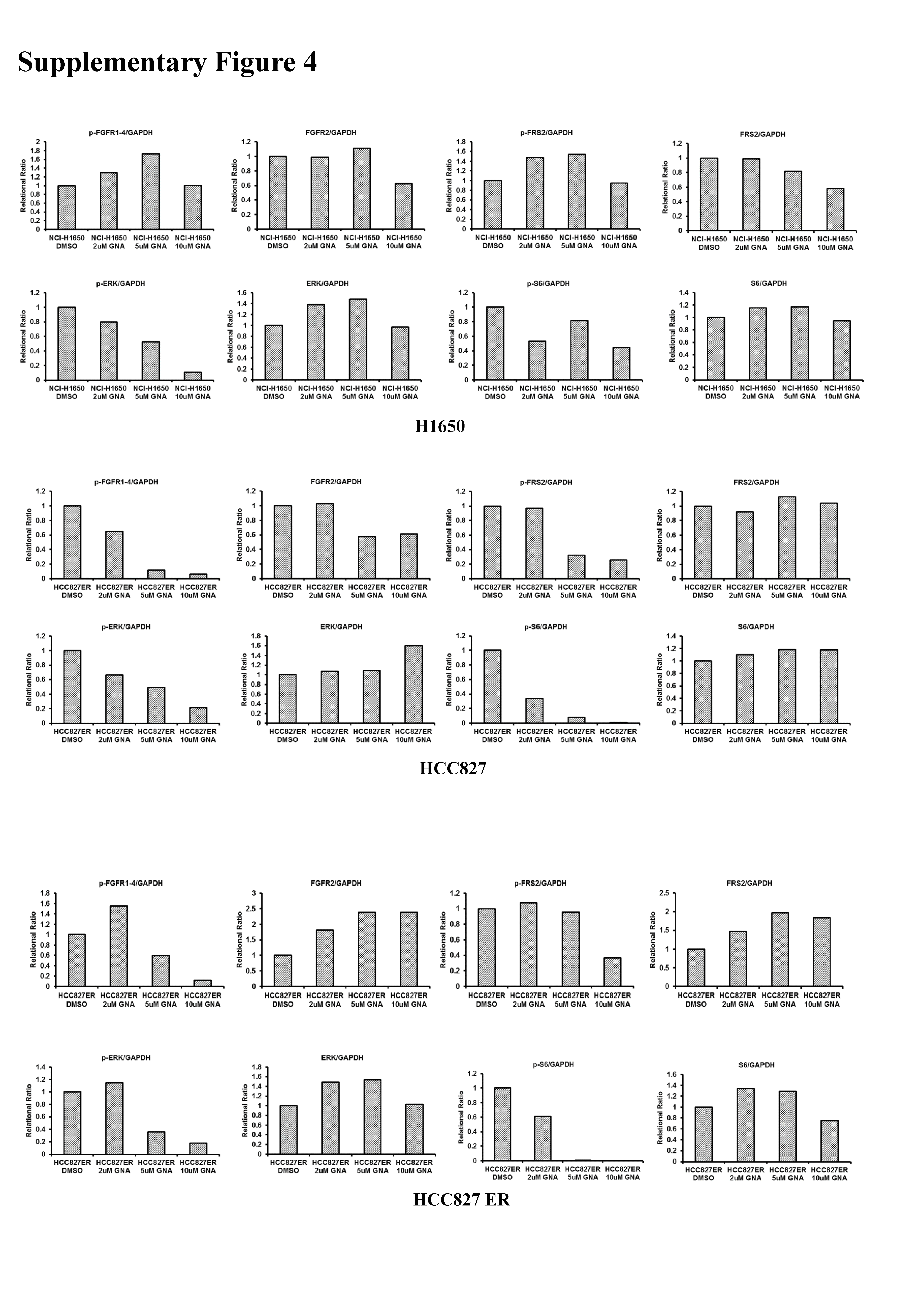

Supplement: Supplementary file 4 — Supplementary Figure 4 [file 41419_2018_314_MOESM4_ESM.tif]

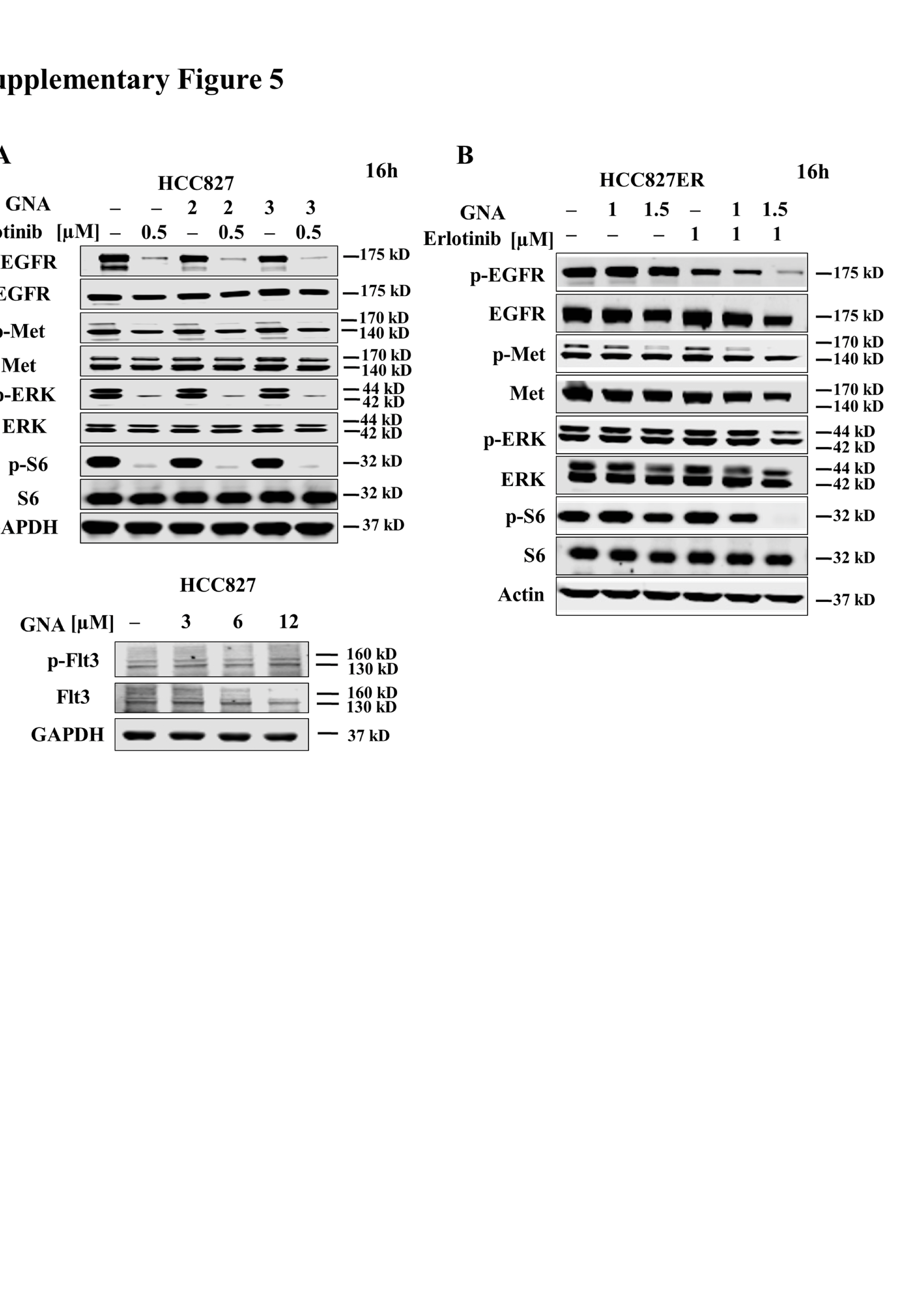

Supplement: Supplementary file 5 — Supplementary Figure 5 [file 41419_2018_314_MOESM5_ESM.tif]

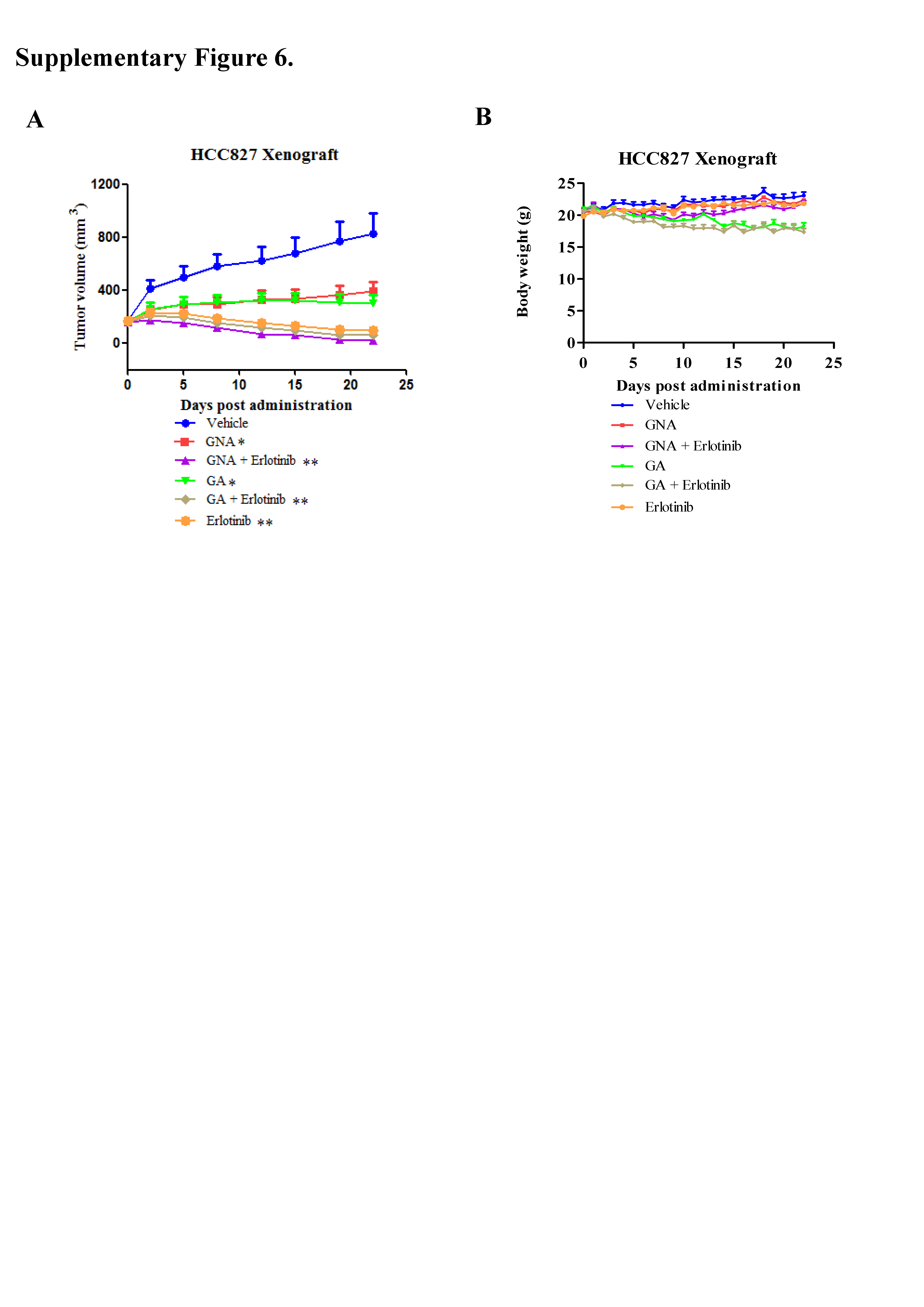

Supplement: Supplementary file 6 — Supplementary Figure 6 [file 41419_2018_314_MOESM6_ESM.tif]

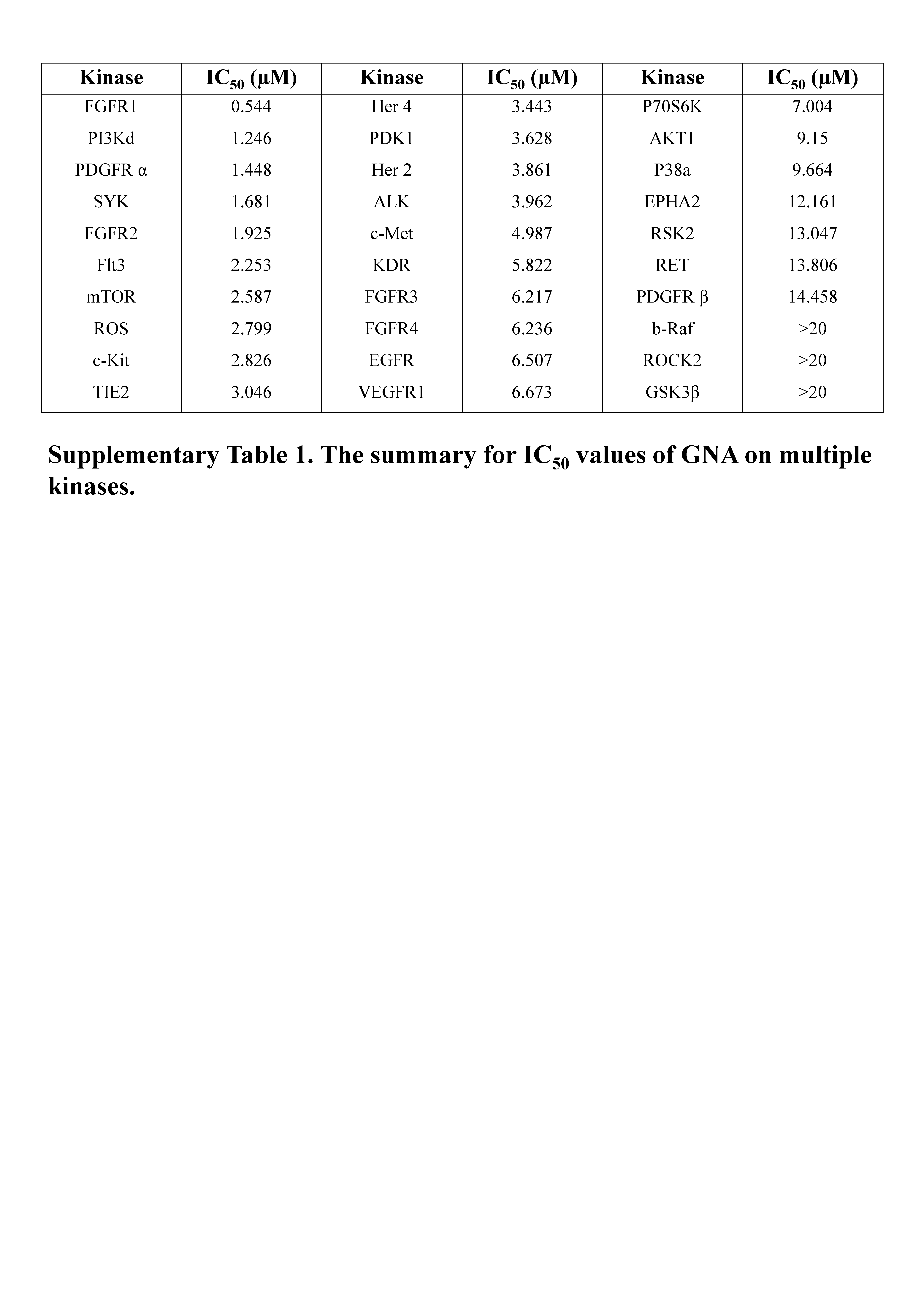

Supplement: Supplementary file 7 — Supplementary Table 1 [file 41419_2018_314_MOESM7_ESM.tif]

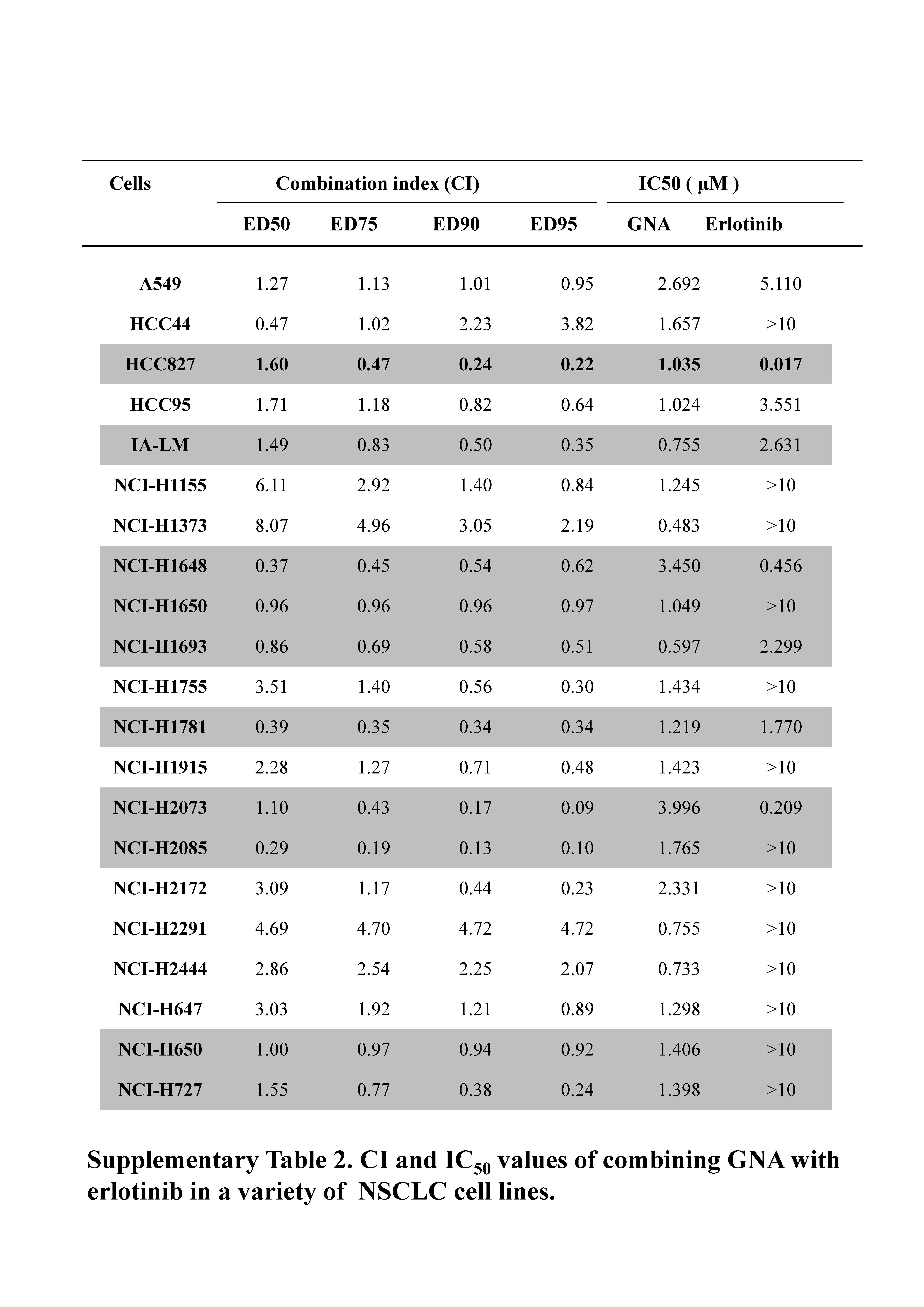

Supplement: Supplementary file 8 — Supplementary Table 2 [file 41419_2018_314_MOESM8_ESM.tif]

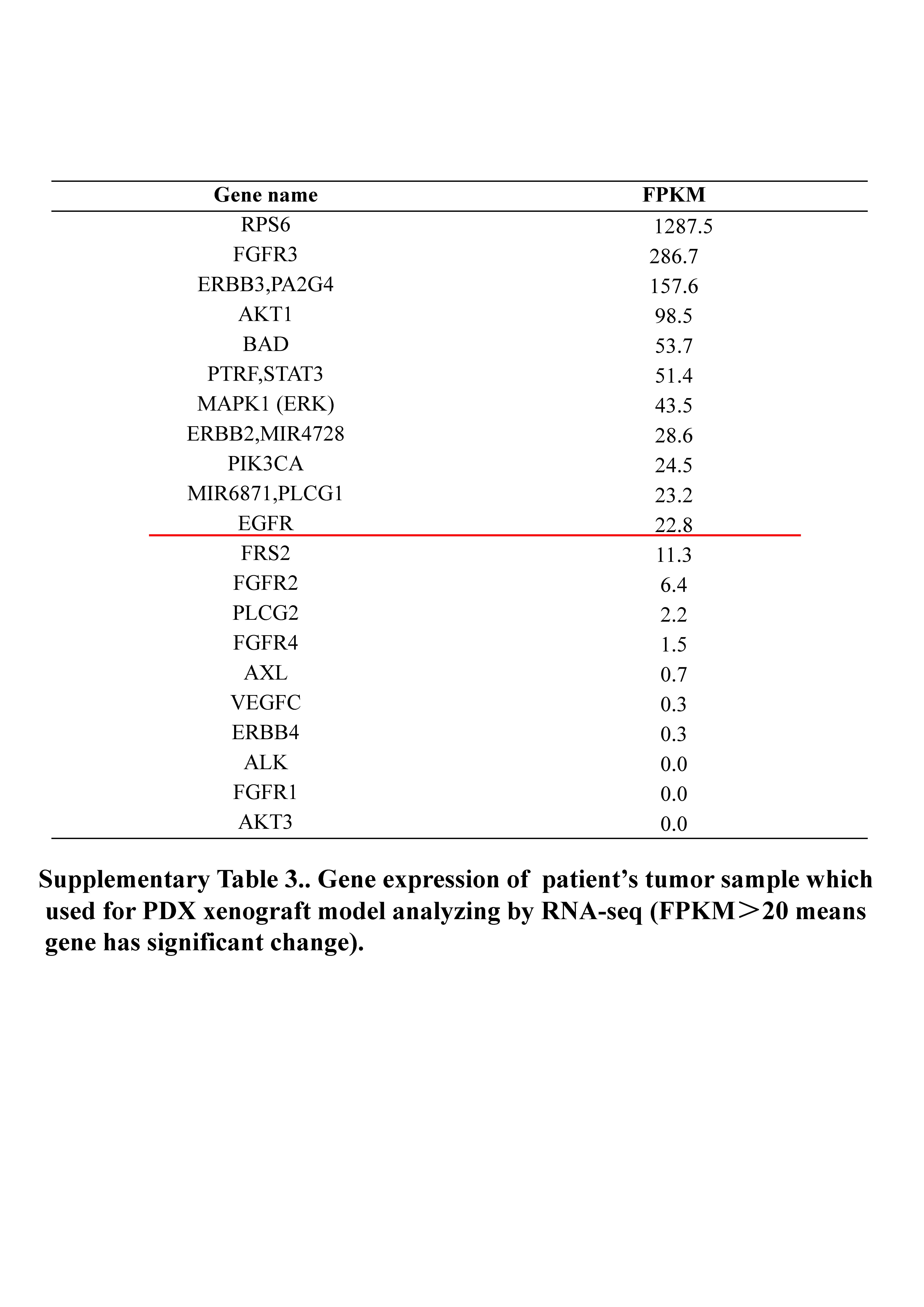

Supplement: Supplementary file 9 — Supplementary Table 3 [file 41419_2018_314_MOESM9_ESM.tif]
